# Photovoltaic properties of ZnO nanorods/p-type Si heterojunction structures

**DOI:** 10.3762/bjnano.5.17

**Published:** 2014-02-14

**Authors:** Rafal Pietruszka, Bartlomiej Slawomir Witkowski, Grzegorz Luka, Lukasz Wachnicki, Sylwia Gieraltowska, Krzysztof Kopalko, Eunika Zielony, Piotr Bieganski, Ewa Placzek-Popko, Marek Godlewski

**Affiliations:** 1 Institute of Physics, Polish Academy of Sciences, Warsaw, Poland; 2 Institute of Physics, WroclawUniversity of Technology, Wroclaw, Poland; 3 Department of Mathematics and Natural Sciences College of Science, Cardinal Stefan Wyszynski University, Warsaw, Poland

**Keywords:** atomic layer deposition, hydrothermal method, solar cells, zinc oxide, zinc oxide nanorods

## Abstract

Selected properties of photovoltaic (PV) structures based on n-type zinc oxide nanorods grown by a low temperature hydrothermal method on p-type silicon substrates (100) are investigated. PV structures were covered with thin films of Al doped ZnO grown by atomic layer deposition acting as transparent electrodes. The investigated PV structures differ in terms of the shapes and densities of their nanorods. The best response is observed for the structure containing closely-spaced nanorods, which show light conversion efficiency of 3.6%.

## Introduction

Solar cells are intensively studied as an alternative energy source and may replace conventional energy sources based on fossil fuels in the future. Since the first photovoltaic (PV) structures were shown by the Bell Laboratories in the 1950s [1], concentrated efforts led to the development of a range of possible PV systems. Nowadays multi-junction photovoltaic structures have an efficiency beyond 40% under laboratory conditions [2–3]. Typical PV structures achieve an efficiency of about 20% for crystalline silicon [4] and about 16% for cadmium telluride [5]. Unfortunately, the high costs of the generated electricity prevents that PV systems are more widely spread. The interest in photovoltaic (PV) structures stems from the fact that solar cells are environmentally friendly, rather than from the low costs of energy production. To reduce these costs, efforts to improve efficiency and a concentrated search for cheaper materials and structures are undertaken.

Wide band gap semiconductors have been studied since the 1930s [6], and several applications have been found in the past decades. For example, they are used in PV systems based on thin films, so-called PV structures of the second generation [7–9]. At the moment, zinc oxide is the most studied wide band gap material [10–14]. ZnO has a 3.37 eV direct band gap at room temperature [15] and a high excitation binding energy of 60 meV. It is intensively studied for light emitters in the near-UV region of the spectrum [16–17], or for spintronic applications [18–20], since ferromagnetic thin films of ZnO can be used to store data for a long period of time [21]. In PV structures, ZnO can replace the commonly used indium tin oxide (ITO) as a transparent electrode. Thin films of ZnO doped with aluminum (ZnO:Al, AZO) or gallium (ZnO:Ga, GZO) obtained by various deposition methods, show a resistivity of the order of 10^−4^ Ω·cm and a high transparency [22–23]. Due to these properties and the low costs of ZnO and deposition methods, ZnO:Al films may be used in PV structures as a replacement for expensive ITO layers [24–26].

One-dimensional (1D) nanostructures such as nanorods attracted a lot of attention due to their ability of being used as building blocks for future electronic, photonic, electromechanical and PV devices [27–32]. Many research groups have been working on various 1D semiconductor systems for the past few years [33–35].

In this work, we study PV structures based on zinc oxide nanorods grown by a hydrothermal method on top of p-type Si, covered on top with ZnO:Al films grown by atomic layer deposition (ALD) and acting as a transparent electrode. These simple and low costs solar cells show a power conversion efficiency, which we consider satisfactory.

## Experimental

**Figure 1:**
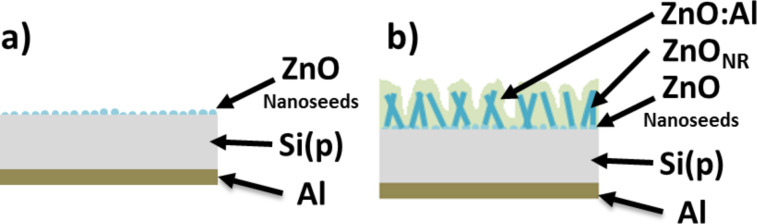
Schematic drawings of the investigated solar cells structure based on zinc oxide nanorods (not to scale).

In this work, we investigate n-type ZnO/p-type Si heterostructures. P-type (100) silicon wafer with a resistance of 2.32 Ω cm was cut into pieces of the size of 0.5 cm^2^. Cut silicon pieces were cleaned in 2-propanol, acetone and deionized water for 5 minutes by using an ultrasonic cleaner. Then, in the ALD process with 15 cycles, ZnO nanoseeds were deposited on a Si substrate (Figure 1a). The deposited ZnO nano-islands nucleate growth of ZnO nanorods in a hydrothermal process, performed in a Ertec01-03 Magnum reactor [36–39]. The growth of the ZnO nanorods was performed at atmospheric pressure and a temperature of about 60 °C.

The reaction mixture was prepared by dissolving zinc acetate in deionized water. Due to the precipitation of sodium hydroxide we obtained a solution with pH values of 7, 7.5 and 8. The pH values in this range control the size and density of nanorods. For example, given a solution with a pH value of 7 the ZnO nanorods have a length of approximately 800 nm and a width of approximately 300 nm. For higher pH values, the length of the nanorods increases their width decreases, and their density increases. For pH 8, the nanorods have a width of approximately 200 nm, a length of approximately 800 nm, and are close to each other. Average sizes of ZnO NRs and ZnO NRs covered ZnO:Al layers are summarized in Table 1. We obtained three distinct types of ZnO nanorods by changing the pH values denoted as sample A, sample B and sample C.

**Table 1:** Average sizes of ZnO NRs and ZnO NRs covered ZnO:Al grown at different pH values.

pH	Average height of ZnO NRs [nm]	Average width of ZnO NRs [nm]	Average height of ZnO:Al/ZnO NRs [nm]	Average width of ZnO:Al/ZnO NRs [nm]

7	800	300	1100	800
7.5	1050	400	1400	800
8	800	200	1150	650

ZnO with aluminum atoms (AZO) were grown on ZnO nanorods (ZnO_NR_)/Si structures by using the ALD process in the Savannah-100 reactor Cambridge NanoTech (Figure 1b) [40–41]. We used diethylzinc (DEZ) and deionized water as zinc and oxygen precursors, respectively. For doping zinc oxide layers trimethylaluminum (TMA) was used as an aluminum precursor. The growth temperature was 160 °C and the N_2_ was used as a purging gas. To obtain a high conductivity of the ZnO film, we mixed ALD cycles. We applied 1 cycle TMA + H_2_O and 24 cycles of DEZ + H_2_O to obtain a uniform distribution of Al in the layer. The lowest resistivity was achieved for ZnO:Al films with 3% of Al. Further details of the ALD growth process are given in our recent publication [42]. Although the growth temperature was relatively low (160 °C), we achieved a metallic-like conductivity. Concentrations, mobilities and resistivities of ZnO:Al films and Si substrates are listed in Table 2. Please note that the low growth temperature – which still facilitates a high conductivity – is important, since we plan to deposit future test devices on transparent foils.

**Table 2:** Electrical parameters of investigated PV structures grown on p Si substrates.

	Thickness	Mobility [cm^2^/Vs]	Concentration [cm^−3^]	Conductivity [Ω^−1^cm^−1^]	Resistivity [Ω·cm]	Carrier type

ZnO:Al	350 nm	11.1	3.33 × 10^20^	629	1.89 × 10^−3^	electrons
Si	270 µm	297	9.08 × 10^15^	0.43	2.32	holes

Aluminum ohmic contacts to p-type Si were deposited by e-beam evaporation (PVD 75, Kurt Lesker). The obtained solar cell structures were characterized by a Scanning Electron Microscope Hitachi SU-70 with an accelerating voltage of 15 kV. PV response was measured by using current–voltage (*I*–*V*) curve tracer for fast *I*–*V* measurements with a sun simulator cl. AAA, at an illumination irradiance of 100 mW/cm^2^. Quantum efficiency was measured by using the PV Quantum efficiency system (EQE and IQE) (300–1100 nm) (Bentham U.K.) and a bias-light.

## Results

**Figure 2:**
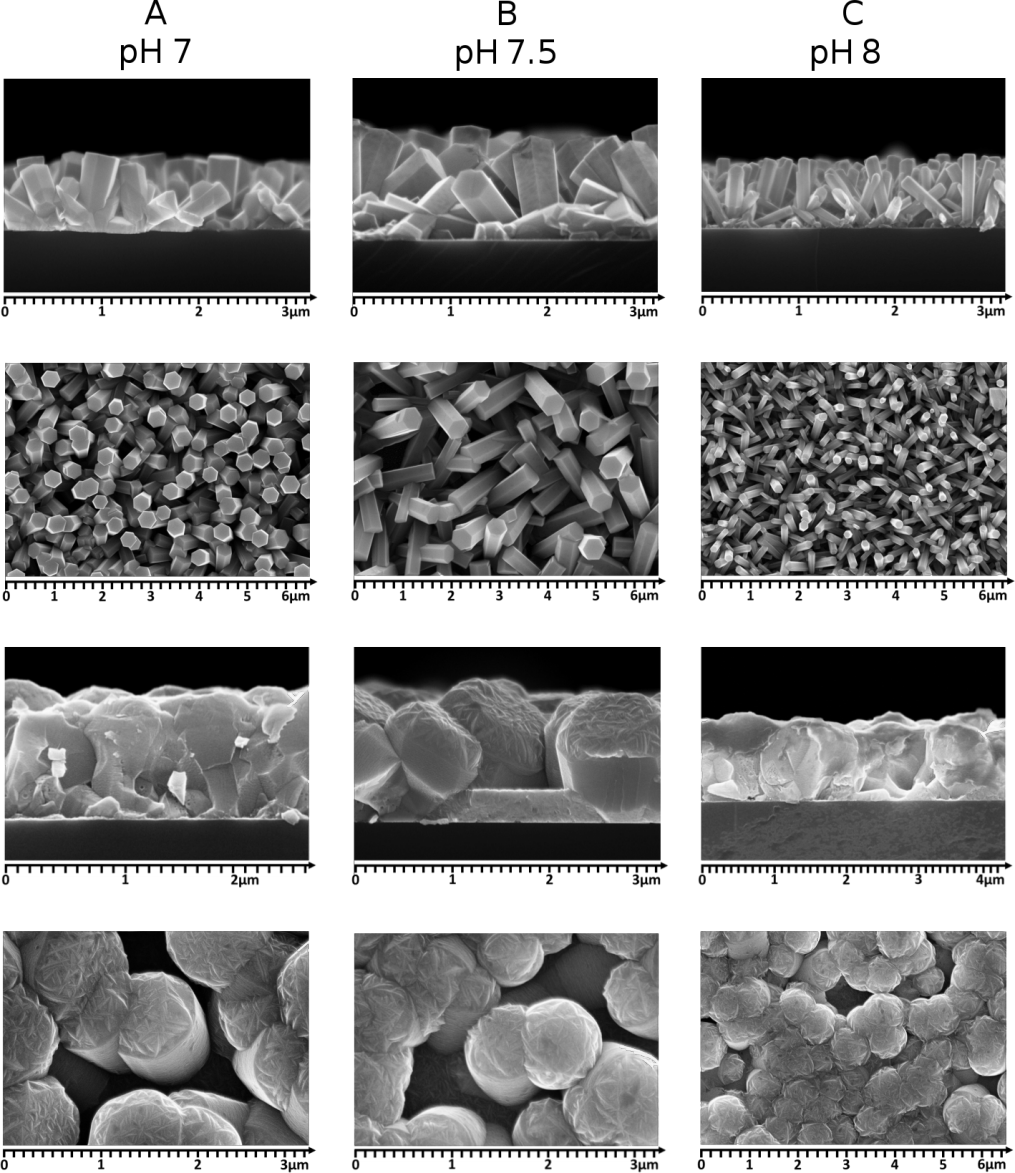
Cross-section and top view (up) SEM images illustrating zinc oxide nanorods grown at different pH values of 7, 7.5 and 8. Images at the bottom show cross-section and top view of ZnONR covered with ZnO:Al layers.

Figure 2 shows scanning electron microscope (SEM) images of three types of the studied ZnO:Al/ZnO_NR_/Si/Al PV structures denoted as A, B and C. Figure 1 (up) shows the cross section and the top view of the nanorods. Figure 2 (down) shows zinc oxide nanorods covered with AZO films. The samples A and B exhibited a similar density of nanorods. For samples A (pH 7) the calculated average length was 800 nm and the width 300 nm. We noticed an increased length and width with an increasing pH value from 7 to 7.5. For samples B (pH 7.5) the length increased from 800 nm to 1050 nm and the width increased from 300 nm to 400 nm. The average density did not change. However, pH values higher than 7.5 lead to decreased lengths and widths. Zinc oxide NRs grown at pH 8 show an average length of 800 nm and a width of 200 nm. For samples C we obtained the highest density of nanorods. The average thickness of ZnO:Al layers was 350 nm. The width changed significantly for samples A and B. The average width for samples A and B is 800 nm and for sample C 650 nm. Different sizes of nanorods enable us to study the influence of ZnO_NR_ on the electrical and optical properties of the ZnO_NR_/Si PV structures.

**Figure 3:**
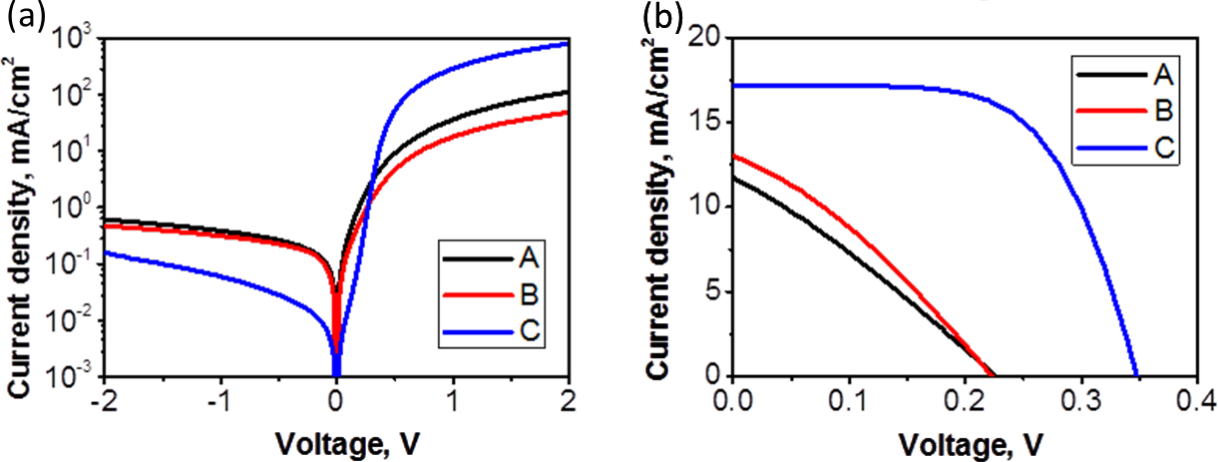
Current–voltage characteristics for the ZnO:Al/ZnO_NR_/Si/Al heterostructures measured under dark (top) and under light conditions (down).

**Figure 4:**
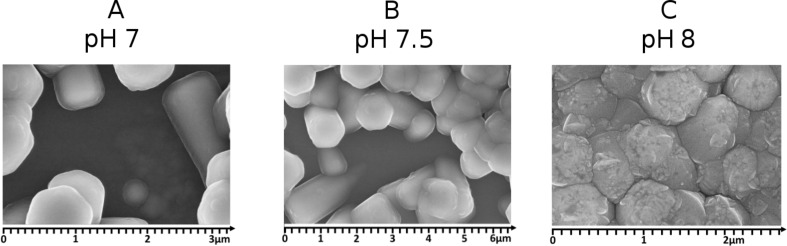
SEM images of the three investigated types of structures with different surface morphologies.

Dark *I*–*V* characteristics of the structures are shown in Figure 3a. From the dark *I*–*V* measurements we see that the size and density of the nanorods affect the diode parameters. Sample A and sample B with similar widths and densities of the nanorods display similar *I*–*V* curves. Rectification ratios calculated at 1 V are about 10^2^ for the samples A and B and 4.7 × 10^3^ for the sample C. For nanorods with a low density (structures A and B) the deposited ZnO:Al films cover not only nanorods but also fill in the gaps between them (Figure 4). Samples A and B show a core-shell structure with the zinc oxide nanorod being the core and the AZO layer being the shell. For closely packed nanorods (sample C) the AZO film is grown only on the top of the nanorods. However, we also observed growths of AZO films between single nanorods. Thus, the sample C is the only one to have a “clean” junction between ZnO_NR_ and the silicon as well as an AZO film acting as a transparent top electrode. The fact that the structure C shows the best junction properties indicates that the quality of ZnO nanorods affects the results.

The most important parameter for a comparison of solar cells is the efficiency (η) of light conversion. The efficiency is defined as the ratio of the energy output from the cells (*P*_max_) to the input energy from the sun (*P*_in_). In this work, the efficiency of the solar cells was calculated by using the equation:

[1]
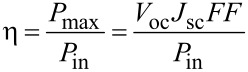


where *V*_oc_ is the open-circuit voltage, *J*_sc_ is the short-circuit current, and *FF* is the fill factor.

The fill factor (*FF*) determines the maximum power from the PV cells. The fill factor is the ratio of the real output from the solar cell (*V*_m_ × *J*_m_) to the product of *V*_oc_ × *J*_sc_. The fill factor can be expressed by the following equation:

[2]
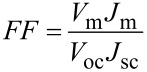


where *V*_m_ is the value of the voltage for the maximum power from a solar cell, and *J*_m_ is the value of the current for the maximum power from a solar cell.

Figure 3b shows *I*–*V* characteristics for the samples A, B and C measured under an illumination of 100 mW/cm^2^. The photovoltaic parameters obtained from the fit are summarized in Table 3. The given data allows the determination of the relationship between *R*_sh_ (shunt resistance), *R*_s_ (series resistance) and the PV response. For the samples A and B the shunt resistance is very low. The low value of the shunt resistance indicates the presence of a low connection of recombination paths for photo-generated electron-hole pairs. The large value of *R*_sh_ reduces the current flowing through the junction and reduces the value of *V*_oc_. The current value is influenced by the structure morphology. Samples A and B show a non-uniform ZnO:Al layer morphology. Probably, this increases the scattering of photo-generated carriers. Sample C with uniform AZO film morphology exhibits the highest value of photo-generated current. The calculated value of *R*_s_ is relatively high for samples A and B. The main impact of *R*_s_ is the reduction of *FF* in the investigated structures. In case of sample C, the best value of *R*_s_ equals 5.1 Ω. We noticed that *FF* decreases from 38% to 28% when *R*_s_ increases from 5.1 Ω to 20.8 Ω. We observe an increase of the short circuit current, open circuit voltage and the filling factor for sample C. Consequently, the best PV response is observed for sample C with a value of 3.6%.

**Table 3:** Photovoltaic parameters for the investigated heterostructures.

No.	*R*_sh_ [Ω]	*R*_s_ [Ω]	*J*_sc_ [mA/cm^2^]	*V*_oc_ [V]	*J*_m_ [mA/cm^2^]	*V*_m_ [V]	*FF* [%]	Efficiency [%]

A	69.8	20.8	12	0.23	6	0.12	28	0.9
B	86.7	11.8	13	0.23	8	0.12	30	1
C	2038	5.1	17	0.35	16	0.22	38	3.6

**Figure 5:**
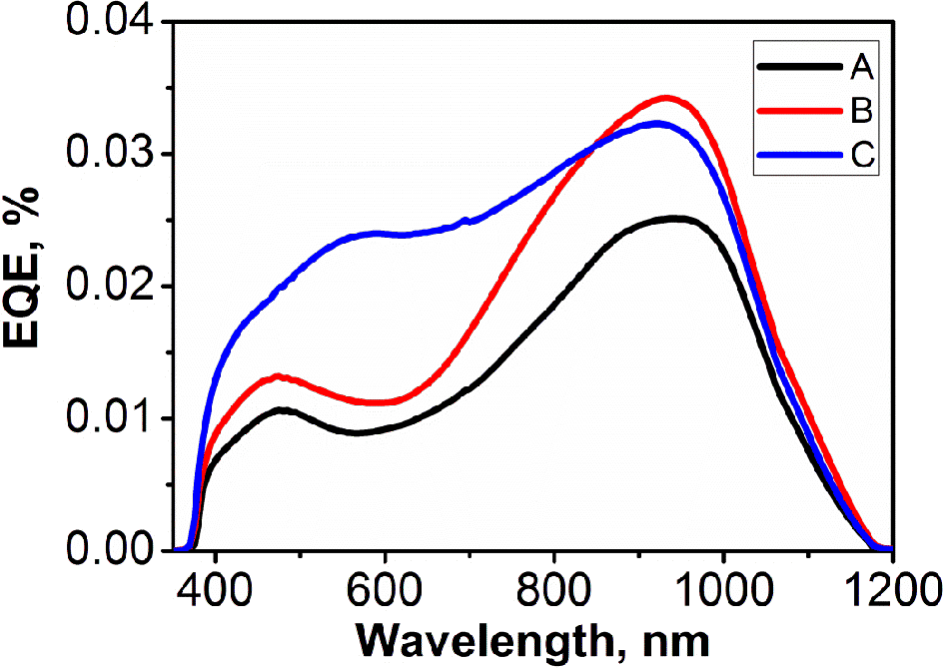
External quantum efficiency of the PV structures of samples A, B and C based on zinc oxide nanorods.

Figure 5 shows the external quantum efficiency measured with an illumination in the range of 350–1200 nm. The investigated PV structures react to the light from 380 nm to 1150 nm. For all samples the highest value of generated photocurrent flows through the junction with an illumination in the range of 900 nm to 1000 nm, i.e., when carriers are generated in a Si substrate. Samples with a similar value of the shunt resistance (A, B) have a similar photoresponse curves. Quantum efficiency values calculated at 950 nm for the samples A, B and C are 2.51 × 10^−2^%, 3.39 × 10^−2^% and 3.17 × 10^−2^%, respectively. The quantum efficiency of structure C, the one with the highest efficiency, is twice as large as for the samples A and B when illuminated with light in the range of 350 nm to 700 nm. This may be explained by the increased absorption of ZnO NRs due to the presence of defect states with energies below the ZnO conduction band gap edge. A similar phenomenon was observed by us in case of a ZnO–organic heterojunction, where an increased absorption in the blue-violet spectra range contributed to a higher PV efficiency of the obtained hybrid PV cells [43]. However, a detailed explanation of the effect observed in this work will require a further study.

## Conclusion

We investigated PV structures based on zinc oxide nanorods. Their 3D morphology leads to an increased light trapping. ZnO_NR_ with different sizes and density were grown on silicon surfaces by the hydrothermal method. AZO films were grown at a low deposition temperature in the ALD process. This method has the potential of scaling up substrate sizes to more than 1 m^2^. A wide spectral range of the absorption (from 350 nm to 1200 nm) was observed. The wide working spectrum and the good junction quality led to 3.6% quantum efficiency, which can be further improved by, e.g., the plasmonic effect, a refinement of the electrical parameters of the AZO films (higher growth temperature), and an optimization of the contacts (the results given here were collected by using a point contact to AZO films). The investigated PV structures are cheap and easily constructed. We used cheap Si substrates, a very efficient and low-cost technology to produce both nanorods and AZO films, and inexpensive precursors of reactions.
